# Supplementary figures and images for: Resolving kinesin stepping: one head at a time (part 2 of 2)
Source: Life Sci Alliance. 2019 Oct 10;2(5):e201900456. doi: 10.26508/lsa.201900456 (PMC6788457; doi:10.26508/lsa.201900456)

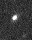

Supplement: Supplementary file 2 [file LSA-2019-00456_Supplement_data_2.zip › raw_data/wt/190321_190227_4_5-3a/190227_4_5-3a.tif]

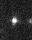

Supplement: Supplementary file 2 [file LSA-2019-00456_Supplement_data_2.zip › raw_data/wt/190321_190227_4_5-3b/190227_4_5-3b.tif]

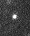

Supplement: Supplementary file 2 [file LSA-2019-00456_Supplement_data_2.zip › raw_data/wt/190321_190227_4_5-4a/190227_4_5-4a.tif]

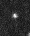

Supplement: Supplementary file 2 [file LSA-2019-00456_Supplement_data_2.zip › raw_data/wt/190321_190227_4_5-4b/190227_4_5-4b.tif]

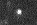

Supplement: Supplementary file 2 [file LSA-2019-00456_Supplement_data_2.zip › raw_data/wt/190321_190227_4_5-6a/190227_4_5-6a.tif]

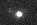

Supplement: Supplementary file 2 [file LSA-2019-00456_Supplement_data_2.zip › raw_data/wt/190321_190227_4_5-6b/190227_4_5-6b.tif]

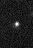

Supplement: Supplementary file 2 [file LSA-2019-00456_Supplement_data_2.zip › raw_data/wt_switched_heads/190812_190808_3_2-2a/190808_3_2-2a.tif]

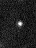

Supplement: Supplementary file 2 [file LSA-2019-00456_Supplement_data_2.zip › raw_data/wt_switched_heads/190812_190808_3_2-2b/190808_3_2-2b.tif]

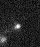

Supplement: Supplementary file 2 [file LSA-2019-00456_Supplement_data_2.zip › raw_data/wt_switched_heads/190812_190808_3_2-4a/190808_3_2-4a.tif]

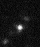

Supplement: Supplementary file 2 [file LSA-2019-00456_Supplement_data_2.zip › raw_data/wt_switched_heads/190812_190808_3_2-4b/190808_3_2-4b.tif]

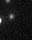

Supplement: Supplementary file 2 [file LSA-2019-00456_Supplement_data_2.zip › raw_data/wt_switched_heads/190812_190808_3_2-9a/190808_3_2-9a.tif]

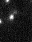

Supplement: Supplementary file 2 [file LSA-2019-00456_Supplement_data_2.zip › raw_data/wt_switched_heads/190812_190808_3_2-9b/190808_3_2-9b.tif]

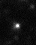

Supplement: Supplementary file 2 [file LSA-2019-00456_Supplement_data_2.zip › raw_data/wt_switched_heads/190812_190808_5_3-7a/190808_5_3-7a.tif]

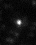

Supplement: Supplementary file 2 [file LSA-2019-00456_Supplement_data_2.zip › raw_data/wt_switched_heads/190812_190808_5_3-7b/190808_5_3-7b.tif]

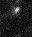

Supplement: Supplementary file 2 [file LSA-2019-00456_Supplement_data_2.zip › raw_data/wt_switched_heads/190812_190808_5_3-9a/190808_5_3-9a.tif]

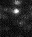

Supplement: Supplementary file 2 [file LSA-2019-00456_Supplement_data_2.zip › raw_data/wt_switched_heads/190812_190808_5_3-9b/190808_5_3-9b.tif]

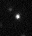

Supplement: Supplementary file 2 [file LSA-2019-00456_Supplement_data_2.zip › raw_data/wt_switched_heads/190812_190808_6_3-2a/190808_6_3-2a.tif]

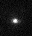

Supplement: Supplementary file 2 [file LSA-2019-00456_Supplement_data_2.zip › raw_data/wt_switched_heads/190812_190808_6_3-2b/190808_6_3-2b.tif]

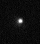

Supplement: Supplementary file 2 [file LSA-2019-00456_Supplement_data_2.zip › raw_data/wt_switched_heads/190812_190808_6_4-1a/190808_6_4-1a.tif]

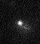

Supplement: Supplementary file 2 [file LSA-2019-00456_Supplement_data_2.zip › raw_data/wt_switched_heads/190812_190808_6_4-1b/190808_6_4-1b.tif]

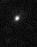

Supplement: Supplementary file 2 [file LSA-2019-00456_Supplement_data_2.zip › raw_data/wt_switched_heads/190812_190808_6_4-4a/190808_6_4-4a.tif]

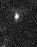

Supplement: Supplementary file 2 [file LSA-2019-00456_Supplement_data_2.zip › raw_data/wt_switched_heads/190812_190808_6_4-4b/190808_6_4-4b.tif]

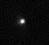

Supplement: Supplementary file 2 [file LSA-2019-00456_Supplement_data_2.zip › raw_data/wt_switched_heads/190812_190808_7_2-1a/190808_7_2-1a.tif]

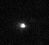

Supplement: Supplementary file 2 [file LSA-2019-00456_Supplement_data_2.zip › raw_data/wt_switched_heads/190812_190808_7_2-1b/190808_7_2-1b.tif]

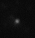

Supplement: Supplementary file 2 [file LSA-2019-00456_Supplement_data_2.zip › raw_data/wt_switched_heads/190812_190809_2_2-1a/190809_2_2-1a.tif]

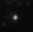

Supplement: Supplementary file 2 [file LSA-2019-00456_Supplement_data_2.zip › raw_data/wt_switched_heads/190812_190809_2_2-1b/190809_2_2-1b.tif]

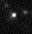

Supplement: Supplementary file 2 [file LSA-2019-00456_Supplement_data_2.zip › raw_data/wt_switched_heads/190812_190809_2_2-2a/190809_2_2-2a.tif]

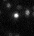

Supplement: Supplementary file 2 [file LSA-2019-00456_Supplement_data_2.zip › raw_data/wt_switched_heads/190812_190809_2_2-2b/190809_2_2-2b.tif]

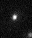

Supplement: Supplementary file 2 [file LSA-2019-00456_Supplement_data_2.zip › raw_data/wt_switched_heads/190812_190809_2_2-3a/190809_2_2-3a.tif]

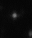

Supplement: Supplementary file 2 [file LSA-2019-00456_Supplement_data_2.zip › raw_data/wt_switched_heads/190812_190809_2_2-3b/190809_2_2-3b.tif]

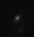

Supplement: Supplementary file 2 [file LSA-2019-00456_Supplement_data_2.zip › raw_data/wt_switched_heads/190812_190809_2_2-4a/190809_2_2-4a.tif]

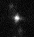

Supplement: Supplementary file 2 [file LSA-2019-00456_Supplement_data_2.zip › raw_data/wt_switched_heads/190812_190809_2_2-4b/190809_2_2-4b.tif]

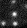

Supplement: Supplementary file 2 [file LSA-2019-00456_Supplement_data_2.zip › raw_data/wt_switched_heads/190812_190809_2_2-5a/190809_2_2-5a.tif]

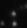

Supplement: Supplementary file 2 [file LSA-2019-00456_Supplement_data_2.zip › raw_data/wt_switched_heads/190812_190809_2_2-5b/190809_2_2-5b.tif]

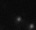

Supplement: Supplementary file 2 [file LSA-2019-00456_Supplement_data_2.zip › raw_data/wt_switched_heads/190812_190809_2_2-7a/190809_2_2-7a.tif]

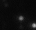

Supplement: Supplementary file 2 [file LSA-2019-00456_Supplement_data_2.zip › raw_data/wt_switched_heads/190812_190809_2_2-7b/190809_2_2-7b.tif]

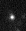

Supplement: Supplementary file 2 [file LSA-2019-00456_Supplement_data_2.zip › raw_data/wt_switched_heads/190812_190809_2_2-8a/190809_2_2-8a.tif]

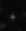

Supplement: Supplementary file 2 [file LSA-2019-00456_Supplement_data_2.zip › raw_data/wt_switched_heads/190812_190809_2_2-8b/190809_2_2-8b.tif]

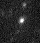

Supplement: Supplementary file 2 [file LSA-2019-00456_Supplement_data_2.zip › raw_data/wt_switched_heads/190812_190809_2_2-9a/190809_2_2-9a.tif]

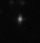

Supplement: Supplementary file 2 [file LSA-2019-00456_Supplement_data_2.zip › raw_data/wt_switched_heads/190812_190809_2_2-9b/190809_2_2-9b.tif]

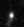

Supplement: Supplementary file 2 [file LSA-2019-00456_Supplement_data_2.zip › raw_data/wt_switched_heads/190814_190808_3_3-8a/190808_3_3-8a.tif]

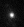

Supplement: Supplementary file 2 [file LSA-2019-00456_Supplement_data_2.zip › raw_data/wt_switched_heads/190814_190808_3_3-8b/190808_3_3-8b.tif]

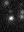

Supplement: Supplementary file 2 [file LSA-2019-00456_Supplement_data_2.zip › raw_data/wt_switched_heads/190814_190809_3_2-3a/190809_3_2-3a.tif]

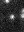

Supplement: Supplementary file 2 [file LSA-2019-00456_Supplement_data_2.zip › raw_data/wt_switched_heads/190814_190809_3_2-3b/190809_3_2-3b.tif]

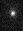

Supplement: Supplementary file 2 [file LSA-2019-00456_Supplement_data_2.zip › raw_data/wt_switched_heads/190814_190809_3_2-4a/190809_3_2-4a.tif]

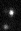

Supplement: Supplementary file 2 [file LSA-2019-00456_Supplement_data_2.zip › raw_data/wt_switched_heads/190814_190809_3_2-4b/190809_3_2-4b.tif]

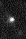

Supplement: Supplementary file 2 [file LSA-2019-00456_Supplement_data_2.zip › raw_data/wt_switched_heads/190814_190809_4_2-4a/190809_4_2-4a.tif]

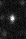

Supplement: Supplementary file 2 [file LSA-2019-00456_Supplement_data_2.zip › raw_data/wt_switched_heads/190814_190809_4_2-4b/190809_4_2-4b.tif]

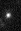

Supplement: Supplementary file 2 [file LSA-2019-00456_Supplement_data_2.zip › raw_data/wt_switched_heads/190814_190809_4_2-6a/190809_4_2-6a.tif]

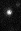

Supplement: Supplementary file 2 [file LSA-2019-00456_Supplement_data_2.zip › raw_data/wt_switched_heads/190814_190809_4_2-6b/190809_4_2-6b.tif]
