# Supplementary figures and images for: Resolving kinesin stepping: one head at a time (part 1 of 2)
Source: Life Sci Alliance. 2019 Oct 10;2(5):e201900456. doi: 10.26508/lsa.201900456 (PMC6788457; doi:10.26508/lsa.201900456)

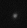

Supplement: Supplementary file 2 [file LSA-2019-00456_Supplement_data_2.zip › raw_data/ee/180821_180814_3_1-3a/180814_3_1-3a.tif]

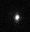

Supplement: Supplementary file 2 [file LSA-2019-00456_Supplement_data_2.zip › raw_data/ee/180821_180814_3_1-3b/180814_3_1-3b.tif]

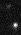

Supplement: Supplementary file 2 [file LSA-2019-00456_Supplement_data_2.zip › raw_data/ee/180821_180814_3_4-10a/180814_3_4-10a.tif]

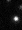

Supplement: Supplementary file 2 [file LSA-2019-00456_Supplement_data_2.zip › raw_data/ee/180821_180814_3_4-10b/180814_3_4-10b.tif]

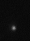

Supplement: Supplementary file 2 [file LSA-2019-00456_Supplement_data_2.zip › raw_data/ee/180821_180814_3_4-1a/180814_3_4-1a.tif]

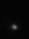

Supplement: Supplementary file 2 [file LSA-2019-00456_Supplement_data_2.zip › raw_data/ee/180821_180814_3_4-1b/180814_3_4-1b.tif]

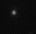

Supplement: Supplementary file 2 [file LSA-2019-00456_Supplement_data_2.zip › raw_data/ee/180821_180814_3_4-2a/180814_3_4-2a.tif]

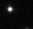

Supplement: Supplementary file 2 [file LSA-2019-00456_Supplement_data_2.zip › raw_data/ee/180821_180814_3_4-2b/180814_3_4-2b.tif]

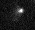

Supplement: Supplementary file 2 [file LSA-2019-00456_Supplement_data_2.zip › raw_data/ee/180821_180814_3_4-8a/180814_3_4-8a.tif]

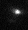

Supplement: Supplementary file 2 [file LSA-2019-00456_Supplement_data_2.zip › raw_data/ee/180821_180814_3_4-8b/180814_3_4-8b.tif]

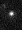

Supplement: Supplementary file 2 [file LSA-2019-00456_Supplement_data_2.zip › raw_data/ee/180822_180814_3_6-3a/180814_3_6-3a.tif]

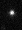

Supplement: Supplementary file 2 [file LSA-2019-00456_Supplement_data_2.zip › raw_data/ee/180822_180814_3_6-3b/180814_3_6-3b.tif]

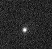

Supplement: Supplementary file 2 [file LSA-2019-00456_Supplement_data_2.zip › raw_data/ee/180828_180814_4_4-4a/180814_4_4-4a.tif]

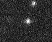

Supplement: Supplementary file 2 [file LSA-2019-00456_Supplement_data_2.zip › raw_data/ee/180828_180814_4_4-4b/180814_4_4-4b.tif]

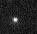

Supplement: Supplementary file 2 [file LSA-2019-00456_Supplement_data_2.zip › raw_data/ee/180829_180814_4_5-4a/180814_4_5-4a.tif]

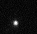

Supplement: Supplementary file 2 [file LSA-2019-00456_Supplement_data_2.zip › raw_data/ee/180829_180814_4_5-4b/180814_4_5-4b.tif]

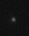

Supplement: Supplementary file 2 [file LSA-2019-00456_Supplement_data_2.zip › raw_data/ee/180829_180814_5_2-4a/180814_5_2-4a.tif]

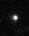

Supplement: Supplementary file 2 [file LSA-2019-00456_Supplement_data_2.zip › raw_data/ee/180829_180814_5_2-4b/180814_5_2-4b.tif]

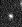

Supplement: Supplementary file 2 [file LSA-2019-00456_Supplement_data_2.zip › raw_data/ee/180829_180814_5_2-9a/180814_5_2-9a.tif]

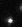

Supplement: Supplementary file 2 [file LSA-2019-00456_Supplement_data_2.zip › raw_data/ee/180829_180814_5_2-9b/180814_5_2-9b.tif]

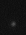

Supplement: Supplementary file 2 [file LSA-2019-00456_Supplement_data_2.zip › raw_data/ee/180829_180814_5_3-5a/180814_5_3-5a.tif]

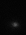

Supplement: Supplementary file 2 [file LSA-2019-00456_Supplement_data_2.zip › raw_data/ee/180829_180814_5_3-5b/180814_5_3-5b.tif]

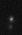

Supplement: Supplementary file 2 [file LSA-2019-00456_Supplement_data_2.zip › raw_data/ee/180829_180814_5_3-7a/180814_5_3-7a.tif]

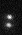

Supplement: Supplementary file 2 [file LSA-2019-00456_Supplement_data_2.zip › raw_data/ee/180829_180814_5_3-7b/180814_5_3-7b.tif]

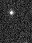

Supplement: Supplementary file 2 [file LSA-2019-00456_Supplement_data_2.zip › raw_data/ee/180829_180814_5_3-9a/180814_5_3-9a.tif]

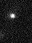

Supplement: Supplementary file 2 [file LSA-2019-00456_Supplement_data_2.zip › raw_data/ee/180829_180814_5_3-9b/180814_5_3-9b.tif]

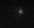

Supplement: Supplementary file 2 [file LSA-2019-00456_Supplement_data_2.zip › raw_data/ee/180829_180814_5_4-9a/180814_5_4-9a.tif]

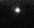

Supplement: Supplementary file 2 [file LSA-2019-00456_Supplement_data_2.zip › raw_data/ee/180829_180814_5_4-9b/180814_5_4-9b.tif]

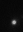

Supplement: Supplementary file 2 [file LSA-2019-00456_Supplement_data_2.zip › raw_data/ee_switched_colors/180822_180814_7_2-1a/180814_7_2-1a.tif]

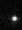

Supplement: Supplementary file 2 [file LSA-2019-00456_Supplement_data_2.zip › raw_data/ee_switched_colors/180822_180814_7_2-1b/180814_7_2-1b.tif]

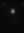

Supplement: Supplementary file 2 [file LSA-2019-00456_Supplement_data_2.zip › raw_data/ee_switched_colors/180822_180814_7_2-6a/180814_7_2-6a.tif]

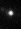

Supplement: Supplementary file 2 [file LSA-2019-00456_Supplement_data_2.zip › raw_data/ee_switched_colors/180822_180814_7_2-6b/180814_7_2-6b.tif]

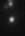

Supplement: Supplementary file 2 [file LSA-2019-00456_Supplement_data_2.zip › raw_data/ee_switched_colors/180822_180814_8_2-1a/180814_8_2-1a.tif]

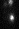

Supplement: Supplementary file 2 [file LSA-2019-00456_Supplement_data_2.zip › raw_data/ee_switched_colors/180822_180814_8_2-1b/180814_8_2-1b.tif]

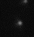

Supplement: Supplementary file 2 [file LSA-2019-00456_Supplement_data_2.zip › raw_data/ee_switched_colors/180822_180814_8_2-4a/180814_8_2-4a.tif]

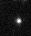

Supplement: Supplementary file 2 [file LSA-2019-00456_Supplement_data_2.zip › raw_data/ee_switched_colors/180822_180814_8_2-4b/180814_8_2-4b.tif]

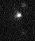

Supplement: Supplementary file 2 [file LSA-2019-00456_Supplement_data_2.zip › raw_data/ee_switched_colors/180828_180814_8_3-3a/180814_8_3-3a.tif]

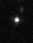

Supplement: Supplementary file 2 [file LSA-2019-00456_Supplement_data_2.zip › raw_data/ee_switched_colors/180828_180814_8_3-3b/180814_8_3-3b.tif]

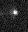

Supplement: Supplementary file 2 [file LSA-2019-00456_Supplement_data_2.zip › raw_data/ee_switched_colors/180828_180814_8_3-4a/180814_8_3-4a.tif]

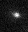

Supplement: Supplementary file 2 [file LSA-2019-00456_Supplement_data_2.zip › raw_data/ee_switched_colors/180828_180814_8_3-4b/180814_8_3-4b.tif]

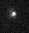

Supplement: Supplementary file 2 [file LSA-2019-00456_Supplement_data_2.zip › raw_data/ee_switched_colors/180828_180814_8_3-6a/180814_8_3-6a.tif]

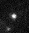

Supplement: Supplementary file 2 [file LSA-2019-00456_Supplement_data_2.zip › raw_data/ee_switched_colors/180828_180814_8_3-6b/180814_8_3-6b.tif]

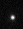

Supplement: Supplementary file 2 [file LSA-2019-00456_Supplement_data_2.zip › raw_data/ee_switched_colors/180828_180814_8_3-9a/180814_8_3-9a.tif]

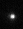

Supplement: Supplementary file 2 [file LSA-2019-00456_Supplement_data_2.zip › raw_data/ee_switched_colors/180828_180814_8_3-9b/180814_8_3-9b.tif]

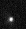

Supplement: Supplementary file 2 [file LSA-2019-00456_Supplement_data_2.zip › raw_data/ee_switched_colors/180828_180814_8_4-4a/180814_8_4-4a.tif]

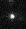

Supplement: Supplementary file 2 [file LSA-2019-00456_Supplement_data_2.zip › raw_data/ee_switched_colors/180828_180814_8_4-4b/180814_8_4-4b.tif]

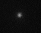

Supplement: Supplementary file 2 [file LSA-2019-00456_Supplement_data_2.zip › raw_data/ee_switched_colors/180828_180814_8_5-4a/180814_8_5-4a.tif]

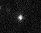

Supplement: Supplementary file 2 [file LSA-2019-00456_Supplement_data_2.zip › raw_data/ee_switched_colors/180828_180814_8_5-4b/180814_8_5-4b.tif]

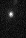

Supplement: Supplementary file 2 [file LSA-2019-00456_Supplement_data_2.zip › raw_data/ee_switched_colors/180828_180814_8_5-5a/180814_8_5-5a.tif]

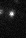

Supplement: Supplementary file 2 [file LSA-2019-00456_Supplement_data_2.zip › raw_data/ee_switched_colors/180828_180814_8_5-5b/180814_8_5-5b.tif]

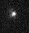

Supplement: Supplementary file 2 [file LSA-2019-00456_Supplement_data_2.zip › raw_data/ee_switched_colors/180830_180814_8_3-6a/180814_8_3-6a.tif]

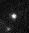

Supplement: Supplementary file 2 [file LSA-2019-00456_Supplement_data_2.zip › raw_data/ee_switched_colors/180830_180814_8_3-6b/180814_8_3-6b.tif]

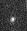

Supplement: Supplementary file 2 [file LSA-2019-00456_Supplement_data_2.zip › raw_data/wt/190107_181218_2_2-1a/181218_2_2-1a.tif]

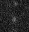

Supplement: Supplementary file 2 [file LSA-2019-00456_Supplement_data_2.zip › raw_data/wt/190107_181218_2_2-1b/181218_2_2-1b.tif]

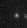

Supplement: Supplementary file 2 [file LSA-2019-00456_Supplement_data_2.zip › raw_data/wt/190107_181218_2_3-1a/181218_2_3-1a.tif]

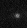

Supplement: Supplementary file 2 [file LSA-2019-00456_Supplement_data_2.zip › raw_data/wt/190107_181218_2_3-1b/181218_2_3-1b.tif]

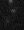

Supplement: Supplementary file 2 [file LSA-2019-00456_Supplement_data_2.zip › raw_data/wt/190107_181218_2_3-5a/181218_2_3-5a.tif]

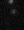

Supplement: Supplementary file 2 [file LSA-2019-00456_Supplement_data_2.zip › raw_data/wt/190107_181218_2_3-5b/181218_2_3-5b.tif]

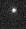

Supplement: Supplementary file 2 [file LSA-2019-00456_Supplement_data_2.zip › raw_data/wt/190107_181218_2_4-1a/181218_2_4-1a.tif]

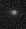

Supplement: Supplementary file 2 [file LSA-2019-00456_Supplement_data_2.zip › raw_data/wt/190107_181218_2_4-1b/181218_2_4-1b.tif]

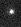

Supplement: Supplementary file 2 [file LSA-2019-00456_Supplement_data_2.zip › raw_data/wt/190107_181218_2_4-2a/181218_2_4-2a.tif]

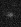

Supplement: Supplementary file 2 [file LSA-2019-00456_Supplement_data_2.zip › raw_data/wt/190107_181218_2_4-2b/181218_2_4-2b.tif]

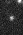

Supplement: Supplementary file 2 [file LSA-2019-00456_Supplement_data_2.zip › raw_data/wt/190107_181218_2_4-3a/181218_2_4-3a.tif]

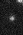

Supplement: Supplementary file 2 [file LSA-2019-00456_Supplement_data_2.zip › raw_data/wt/190107_181218_2_4-3b/181218_2_4-3b.tif]

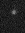

Supplement: Supplementary file 2 [file LSA-2019-00456_Supplement_data_2.zip › raw_data/wt/190107_181218_2_5-1a/181218_2_5-1a.tif]

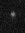

Supplement: Supplementary file 2 [file LSA-2019-00456_Supplement_data_2.zip › raw_data/wt/190107_181218_2_5-1b/181218_2_5-1b.tif]

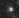

Supplement: Supplementary file 2 [file LSA-2019-00456_Supplement_data_2.zip › raw_data/wt/190107_181218_2_5-9a/181218_2_5-9a.tif]

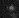

Supplement: Supplementary file 2 [file LSA-2019-00456_Supplement_data_2.zip › raw_data/wt/190107_181218_2_5-9b/181218_2_5-9b.tif]

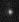

Supplement: Supplementary file 2 [file LSA-2019-00456_Supplement_data_2.zip › raw_data/wt/190108_181218_2_6-2a/181218_2_6-2a.tif]

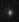

Supplement: Supplementary file 2 [file LSA-2019-00456_Supplement_data_2.zip › raw_data/wt/190108_181218_2_6-2b/181218_2_6-2b.tif]

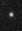

Supplement: Supplementary file 2 [file LSA-2019-00456_Supplement_data_2.zip › raw_data/wt/190108_181218_2_6-6a/181218_2_6-6a.tif]

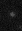

Supplement: Supplementary file 2 [file LSA-2019-00456_Supplement_data_2.zip › raw_data/wt/190108_181218_2_6-6b/181218_2_6-6b.tif]

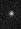

Supplement: Supplementary file 2 [file LSA-2019-00456_Supplement_data_2.zip › raw_data/wt/190108_181218_2_7-1a/181218_2_7-1a.tif]

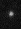

Supplement: Supplementary file 2 [file LSA-2019-00456_Supplement_data_2.zip › raw_data/wt/190108_181218_2_7-1b/181218_2_7-1b.tif]

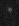

Supplement: Supplementary file 2 [file LSA-2019-00456_Supplement_data_2.zip › raw_data/wt/190108_181218_2_7-2a/181218_2_7-2a.tif]

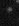

Supplement: Supplementary file 2 [file LSA-2019-00456_Supplement_data_2.zip › raw_data/wt/190108_181218_2_7-2b/181218_2_7-2b.tif]

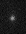

Supplement: Supplementary file 2 [file LSA-2019-00456_Supplement_data_2.zip › raw_data/wt/190108_181218_2_7-6a/181218_2_7-6a.tif]

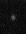

Supplement: Supplementary file 2 [file LSA-2019-00456_Supplement_data_2.zip › raw_data/wt/190108_181218_2_7-6b/181218_2_7-6b.tif]

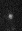

Supplement: Supplementary file 2 [file LSA-2019-00456_Supplement_data_2.zip › raw_data/wt/190108_181218_2_8-1a/181218_2_8-1a.tif]

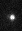

Supplement: Supplementary file 2 [file LSA-2019-00456_Supplement_data_2.zip › raw_data/wt/190108_181218_2_8-1b/181218_2_8-1b.tif]

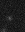

Supplement: Supplementary file 2 [file LSA-2019-00456_Supplement_data_2.zip › raw_data/wt/190109_181218_2_9-6a/181218_2_9-6a.tif]

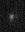

Supplement: Supplementary file 2 [file LSA-2019-00456_Supplement_data_2.zip › raw_data/wt/190109_181218_2_9-6b/181218_2_9-6b.tif]

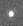

Supplement: Supplementary file 2 [file LSA-2019-00456_Supplement_data_2.zip › raw_data/wt/190214_190211_3_2-2a/190211_3_2-2a.tif]

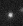

Supplement: Supplementary file 2 [file LSA-2019-00456_Supplement_data_2.zip › raw_data/wt/190214_190211_3_2-2b/190211_3_2-2b.tif]

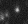

Supplement: Supplementary file 2 [file LSA-2019-00456_Supplement_data_2.zip › raw_data/wt/190228_190227_2_2-1a/190227_2_2-1a.tif]

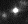

Supplement: Supplementary file 2 [file LSA-2019-00456_Supplement_data_2.zip › raw_data/wt/190228_190227_2_2-1b/190227_2_2-1b.tif]

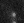

Supplement: Supplementary file 2 [file LSA-2019-00456_Supplement_data_2.zip › raw_data/wt/190228_190227_2_2-4a/190227_2_2-4a.tif]

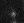

Supplement: Supplementary file 2 [file LSA-2019-00456_Supplement_data_2.zip › raw_data/wt/190228_190227_2_2-4b/190227_2_2-4b.tif]

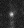

Supplement: Supplementary file 2 [file LSA-2019-00456_Supplement_data_2.zip › raw_data/wt/190228_190227_2_3-1a/190227_2_3-1a.tif]

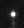

Supplement: Supplementary file 2 [file LSA-2019-00456_Supplement_data_2.zip › raw_data/wt/190228_190227_2_3-1b/190227_2_3-1b.tif]

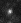

Supplement: Supplementary file 2 [file LSA-2019-00456_Supplement_data_2.zip › raw_data/wt/190228_190227_2_3-2a/190227_2_3-2a.tif]

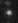

Supplement: Supplementary file 2 [file LSA-2019-00456_Supplement_data_2.zip › raw_data/wt/190228_190227_2_3-2b/190227_2_3-2b.tif]

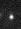

Supplement: Supplementary file 2 [file LSA-2019-00456_Supplement_data_2.zip › raw_data/wt/190228_190227_2_3-4a/190227_2_3-4a.tif]

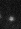

Supplement: Supplementary file 2 [file LSA-2019-00456_Supplement_data_2.zip › raw_data/wt/190228_190227_2_3-4b/190227_2_3-4b.tif]

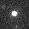

Supplement: Supplementary file 2 [file LSA-2019-00456_Supplement_data_2.zip › raw_data/wt/190301_190227_4_4-2a/190227_4_4-2a.tif]

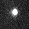

Supplement: Supplementary file 2 [file LSA-2019-00456_Supplement_data_2.zip › raw_data/wt/190301_190227_4_4-2b/190227_4_4-2b.tif]

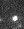

Supplement: Supplementary file 2 [file LSA-2019-00456_Supplement_data_2.zip › raw_data/wt/190301_190227_4_4-4a/190227_4_4-4a.tif]

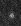

Supplement: Supplementary file 2 [file LSA-2019-00456_Supplement_data_2.zip › raw_data/wt/190301_190227_4_4-4b/190227_4_4-4b.tif]

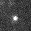

Supplement: Supplementary file 2 [file LSA-2019-00456_Supplement_data_2.zip › raw_data/wt/190301_190227_4_4-9a/190227_4_4-9a.tif]

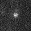

Supplement: Supplementary file 2 [file LSA-2019-00456_Supplement_data_2.zip › raw_data/wt/190301_190227_4_4-9b/190227_4_4-9b.tif]
